# The Relationship Between Physical Exercise and Subjective Well-Being in College Students: The Mediating Effect of Body Image and Self-Esteem

**DOI:** 10.3389/fpsyg.2021.658935

**Published:** 2021-05-28

**Authors:** Yao Shang, Hao-Dong Xie, Shi-Yong Yang

**Affiliations:** ^1^Faculty of Athletics and Swimming, Chengdu Sports University, Chengdu, China; ^2^Research Centre for Exercise Detoxification, College of Physical Education, Southwest University, Chongqing, China

**Keywords:** physical exercise, subjective well-being, body image, self-esteem, college students

## Abstract

This research examines the relationship between physical exercise and subjective well-being *via* the mediation of body image and self-esteem, thereby providing some suggestions on the improvement of subjective well-being in college students. A total of 671 college students from three universities of science and engineering in Sichuan, China voluntarily participated in the survey. Descriptive statistics, Pearson’s product-moment correlation, and mediation model analysis were conducted using the SPSS statistics 19.0. The results showed that (1) the physical exercise level was positively and significantly correlated with the subjective well-being level in each dimension (*R* = 0.12–0.64, *p* < 0.01) (2) college students with the medium and high level of exercise have higher subjective well-being than those with the low level of exercise, and (3) body image and self-esteem played a complete mediation role between physical exercise and subjective well-being. The mediation analysis revealed two paths: first, the single mediating path *via* self-esteem [indirect effect = 0.087, 95% CI: (0.037, 0.141)] and second, the serial mediating path *via* body image and self-esteem [indirect effect = 0.038, 95% CI: (0.021, 0.158)]. Some practical implications have been discussed on the physical exercise intervention for promoting the subjective well-being level in college students.

## Introduction

Physical exercise is a physical activity with a certain intensity, frequency, and duration to improve health (Jiang et al., 2009). As an important means to promote mental health, people pay more and more attention to the role of physical exercise. So far, many researchers have conducted relevant research on the topics and confirmed the relationship between physical exercise and certain mental health indicators, such as emotion, personality, self-concept, and cognitive process (Li et al., 2003; Yin and Fu, 2004). Subjective well-being in the cognitive evaluation of people with their own life satisfaction according to their own standards is an important comprehensive psychological index to measure life quality, such as life satisfaction, positive emotions, or negative emotions (Suh et al., 1998). With the development of positive psychology, people realize that improving subjective well-being is an important way to promote health development (Gao et al., 2015). However, more recent studies showed that contemporary Chinese college students had a low level of subjective well-being (Wang et al., 2017a). Accordingly, this research hopes to clarify the relationship between physical exercise and subjective well-being and to provide a reference for promoting the mental health of college students.

Exercise psychology has confirmed that subjective well-being in college students is closely related to their exercise persistence (Diener, 2000). Chen and Yu (2015) pointed out that physical exercise can make individuals obtain physical and mental satisfaction, improve their subjective evaluation of satisfaction with the quality of life, promote pleasant and optimistic positive emotions, and enhance overall assessment of the quality of life of individuals. Empirical research also proves that joyful, smooth, and peak emotional effects are experienced by participating in physical exercise that can directly improve the subjective well-being of participants (Chen et al., 2013). Therefore, this study puts forward

Hypothesis H1: Physical exercise of college students is positively correlated with subjective well-being.

Body image refers to the image formed by an individual of his/her own body, which is the objective cognition and subjective evaluation of his/her own physical characteristics. It is composed of multiple dimensions, such as appearance, body shape, physical fitness, and health (Wang et al., 2017b). The previous survey showed that owing to many years of exam-oriented education, cognition of physical and mental health of Chinese college students is on the surface, and good self-concept training is ignored. About 54% of college students are not very satisfied with their weight and physical fitness (Wu and Zhang, 2003). Physical appearance has an important influence on first impressions, friendship, and other life issues (Cash, 2004), which suggest that body image may have an important contribution to subjective well-being. The findings by Zhang (2007) showed that among college students, physical self-perception is as important as academic self-perception for life satisfaction. At the same time, physical exercise has a good effect on the body image disorder of college students (Wu and Zhang, 2003). Studies have shown that the body image of individuals who exercise regularly can be corrected (Hausenblas and Fallon, 2006). Therefore, body image may be an important “bridge” for physical exercise to affect subjective happiness. Accordingly, this research proposes

Hypothesis H2: Body image plays a mediating role between physical exercise and the subjective well-being in college students.

Self-esteem is an emotional assessment of the positive or negative self-esteem of an individual and is an important factor predicting the implementation of healthy behaviors (Hu, 2017). Previous studies have shown that self-esteem can reduce depression (Li et al., 2015), improve interpersonal relationships (Murray et al., 2000), and life satisfaction (Diener, 2009). It shows that self-esteem is an important protective factor for individual well-being and one of the best indicators of subjective well-being. The research of Guo (2019) also pointed out that self-esteem reflects self-confidence of individuals in their own abilities, importance, sense of value, and sense of success, reflected the evaluation of the self of an individual, and can well predict subjective well-being. Moreover, researchers have reached a consensus that physical exercise, whether it is extracurricular physical exercise (Yan et al., 2019) or curricular activities (Zhu et al., 2010), can effectively improve the self-esteem of students. Accordingly, this research proposes

Hypothesis H3: Self-esteem plays a mediating role between physical exercise and the subjective well-being of college students.

Many studies have shown that the body image of college students is closely related to their self-esteem. Borges et al. (2010) pointed out that excessive attention of undergraduates to their body shape, coupled with the excessive publicity in media of their ideal body shape, will lead to the formation of low self-esteem. And a study (Ouyang et al., 2019) on 1,000 college students found that a positive body image among college students can enhance self-esteem and form a stable sense of it. Accordingly, this research proposes

Hypothesis H4: Body image and self-esteem play a serial mediating role between physical exercise and subjective well-being of college students.

In summary, physical exercise has a good effect on improving the body image and self-esteem of college students; students with positive body image tend to experience subjective well-being, and self-esteem is an important protective factor for individual well-being; and body image among college students can enhance self-esteem. It can be seen that body image and self-esteem are likely to play a mediating role between physical exercise and the subjective well-being of college students. However, few researchers in previous studies have explored the relationship between physical exercise, body image, self-esteem, and subjective well-being.

**Figure 1:**
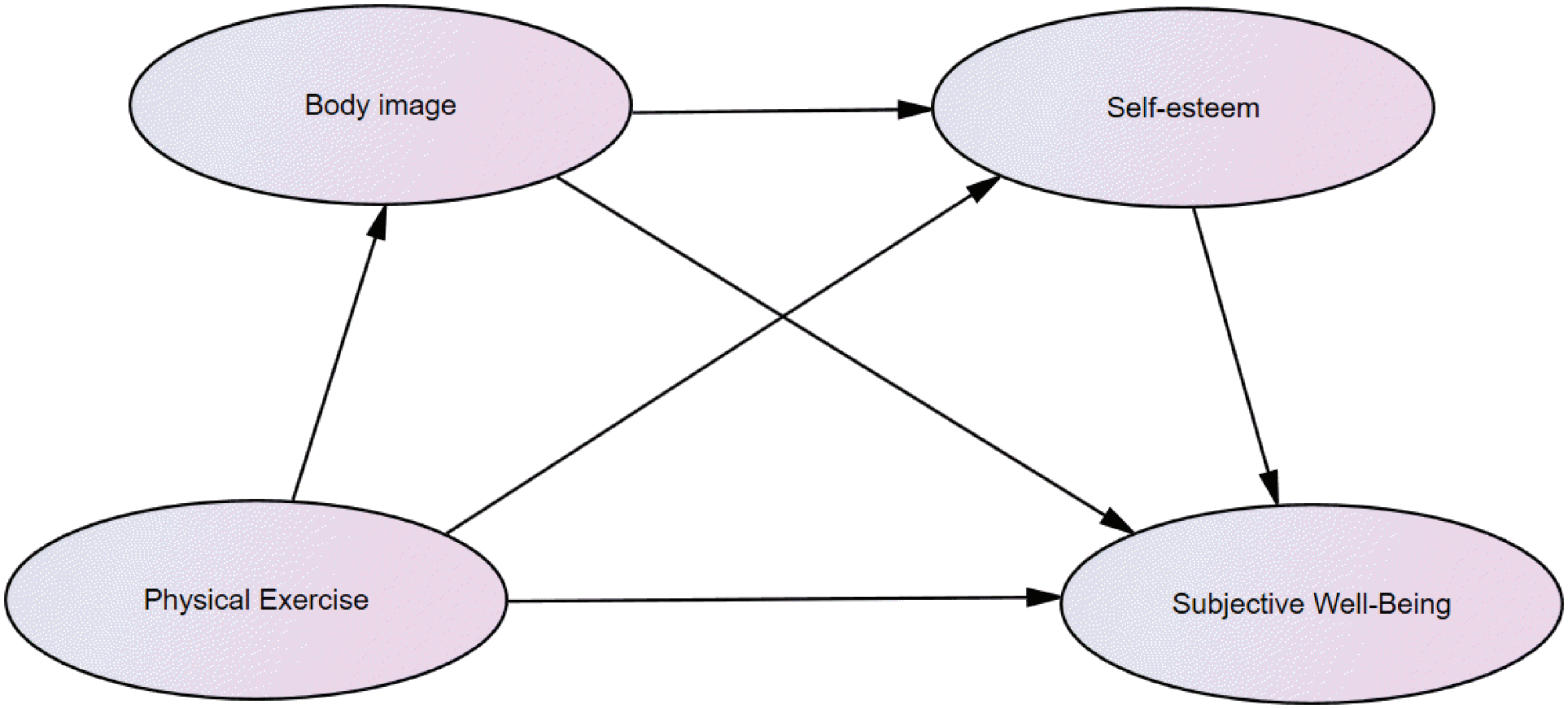
Hypotheses model.

Particularly, there is a lack of in-depth studies on the serial mediating mechanism of “body image-self-esteem.” Based on this, this study intends to use Chinese college students as subjects to observe the relationship between physical exercise and subjective well-being of college students, and also the mediating effect of body image and self-esteem (Figure 1), to provide theoretical bases for further explaining the relationship between physical exercise and subjective well-being and to provide ideas for the improvement of subjective well-being in college students.

## Materials and Methods

### Participants

A sample of 688 undergraduate students from Sichuan in China was recruited in this study as participants by communicating with their counselors *via* email. The sample size is based on the rule of thumb. Each participant filled out the survey questionnaire online according to the instruction ahead of the scale and their counselor from December 12, 2019 to January 21, 2020. After completing the survey, the data preparation was carried out. Seventeen copies were incomplete or had missing data, which have been excluded. However, 671 copies were valid. The valid rate was 97.5%. For more information on the participants, refer to the sociodemographic information as listed in Table 1.

**Table 1:** Summary of the sociodemographic information of the respondents.

Category		Frequency	Percent
Gender			
Female		150	22.4
Male		521	77.6
Origin			
Rural		473	70.5
Urban		198	29.5
Education level			
Freshman		435	64.8
Sophomore		185	27.6
Junior		51	7.6

### Procedure

We began the data collection process by sending emails to 12 counselors from three universities of science and engineering in Sichuan, China. The email detailed the study objectives and the terms of participation (voluntary, anonymous, and confidential). It also included a link to an online platform that contained the measurement instruments items, the instructions for responding to them, and a declaration of informed consent, in accordance with the ethical principles of the Ethics Committee at the Chengdu Sports University and the Declaration of Helsinki. We asked the counselors to distribute the information in the email to the undergraduate students. Thus, the study included those students who, after accepting the above conditions, responded to the questions *via* the online platform, with no time limit.

### Instruments

#### The Personal Background Information

This component covers the basic background information on the gender, education level, and origin of the subject.

#### Physical Activity Rating Scale

The physical activity rating scale (PARS-3) was introduced and revised by Liang (1994) of Wuhan Sports University in China and was widely applied by researchers to measure the level of physical exercise (Liu, 2020). The amount of physical exercise is investigated from three aspects: the intensity of physical exercise, the time of one exercise, and the frequency of one exercise. The level of physical exercise is measured by the amount of physical exercise (Chen and Ji, 2006). A five-point Likert-type scale ranging from 1 to 5 was used. The following formula was used to obtain the total score: score of exercise intensity × (score of exercise time–1) × score of exercise frequency. The standard for the exercise volume was as follows: small exercise volume was ≤19 points; medium exercise volume was 20–42 points; and large exercise volume was ≥43 points. A study on 1,016 college students from China by Ding et al. (2016) showed that its Cronbach’s α coefficient was 0.856. This research showed that the Cronbach’s α coefficient was 0.83 for PARS-3.

#### General Well-Being Schedule

The general well-being schedule (GWB) was compiled by the National Center for Health Statistics of the United States and revised by Duan (1996), a Chinese researcher. The correlation between the single-item score and the total score of the modified scale was between 0.48 and 0.78, and the correlation between the subscale and the total scale was 0.56 and 0.88, and the test-retest consistency was 0.85 (Bao et al., 2010). Referring to the previous explanations of the concept of subjective well-being (Suh et al., 1998), this study selected nine items from three dimensions of GWB to measure subjective well-being of college students: satisfaction and interest in life, for example, “Is your life happy, contented, or enjoyable?”; melancholy or happy mood, for example, “Do you feel depressed and melancholy?”; and relaxation and tension, for example, “How relaxed or tense do you feel?” The total score of subjective well-being is the sum of the scores of nine items. The higher the score, the higher the subjective well-being level. The Cronbach’s α coefficient for the overall GWB was 0.73 (satisfaction and interest in life = 0.68, melancholy or happy mood = 0.68, and relaxation and tension = 0.62).

#### Multidimensional Body Self-Relations Questionnaire

Multidimensional body self-relations questionnaire (MBSRQ) was compiled by Cash et al. (1990) and revised by Song (2016), a Chinese researcher. The internal consistency of each dimension of the modified scale is between 0.7 and 0.8; The verification results of the measurement model were *χ*^2^/*DF* = 1.20, and the values of the comparative fit index (CFI), incremental fit index (IFI), goodness-of-fit index (GFI), and root mean square error of approximation (RMSEA) were 0.908, 0.917, 0.935, and 0.059, respectively. It showed that the scale structure had good validity. In this study, a total of 15 items from three dimensions related to appearance were selected to measure the physical intention of college students: weight concern, for example, “I am on a diet to lose weight”; appearance adaptation, for example, “I will specially organize my hair”; and appearance evaluation, for example, “I like my appearance.” The five-point Likert-type scale from 5 (completely consistent) to 1 (completely inconsistent) was applied. The higher the score, the more positive the body image. The Cronbach’s α coefficient was 0.86 for overall MBSRQ. For the subscales, the Cronbach’s α coefficient was 0.81 for weight concern, 0.91 for appearance evaluation, and 0.91 for appearance adaptation, respectively.

#### The Self-Esteem Scale

The self-esteem scale (SES) compiled by Rosenberg (Tian, 2006) was used to test the level of self-esteem of the college students. It contains a total of 10 items, including two dimensions: self-affirmation, for example, “I feel I have many good qualities” and self-denial, for example, “I am optimistic about myself.” The four-point Likert-type scale from 4 (completely agree) to 1 (completely disagree) was used. The 3rd, 5th, 8th, 9th, and 10th items were reverse coded items. The total score of self-esteem was the sum of all 10 items. Chinese researchers Ouyang et al. (2019) found that the internal consistency of the Cronbach α coefficients of each dimension of the scale was 0.78 and 0.74, respectively. The verification results of the measurement model were *χ*^2^/*DF* = 1.639, and the values of the adjusted goodness-of-fit index (AGFI), CFI, Tucker-Lewis index (TLI), incremental fit index (IFI), GFI, and root mean square error of approximation (RMSEA) were 0.943, 0.991, 0.980, 0.991, 0.979, and 0.051, respectively. It showed that the scale structure had good validity. This research showed that the Cronbach’s α coefficient was 0.74 for overall SES. For the subscales, the Cronbach’s α coefficient was 0.86 for self-affirmation, and 0.72 for self-denial, respectively.

#### Statistical Analyses

Collected data were analyzed using the SPSS 19.0 (IBM Corp., Armonk, NY, United States) and the PROCESS 3.5 macro program developed by Hayes (2013) from University of Calgary, Calgary, Alberta, Canada. First, according to the central limit theorem, data were considered approximately for normal distribution. Second, the internal consistency for each measure was checked by a reliability test using the Cronbach’s α coefficient. Third, the Pearson’s correlation analysis was conducted to examine the relationship between all variables. Fourth, the one-way ANOVA was used to test the difference in the subjective well-being of college students under different levels of physical exercise. Fifth, the regression analysis and process macro program were used for the mediation analysis. The significance level of all tests was set to *α* = 0.05.

## Results

As shown in Table 2, physical exercise, body image, and self-esteem were significantly associated with all dimensions of subjective well-being (*R* = 0.12–0.64). Moreover, there was a significant correlation among physical exercise, body image, and self-esteem. The significant correlation between research variables provides a good foundation for subsequent research hypotheses and mediation testing.

**Table 2:** Descriptive statistics and the Pearson correlation coefficients across physical exercise, body image, self-esteem, and each dimension of subjective well-being in college students (*N* = 671).

Variable	PARS	MBSRQ	SES	GWB	SIL	MHM	RT
PARS	1						
MBSRQ	0.22^***^	1					
SES	0.20^***^	0.31^***^	1				
GWB	0.15^***^	0.25^***^	0.64^***^	1			
SIL	0.14^***^	0.23^***^	0.51^***^	0.55^***^	1		
MHM	0.12^**^	0.20^***^	0.59^***^	0.91^***^	0.35^***^	1	
RT	0.12^**^	0.19^***^	0.50^***^	0.91^***^	0.28^***^	0.76^***^	1
Mean	19.82	39.17	28.92	45.00	6.73	18.05	20.22
SD	21.87	10.75	3.92	7.40	1.94	3.26	3.63

PARS, physical exercise; MBSRQ, body image; SES, self-esteem; SIL, satisfaction and interest in life; MHM, melancholy or happy mood; RT, relaxation and tension.

^**^p < 0.01; ^***^p < 0.001.

In order to more clearly observe the impact of levels of physical activity on subjective well-being, the one-way ANOVA was used to test the difference in the subjective well-being of college students in each exercise group. The study passed the “single-factor homogeneity test” and can use the one-way ANOVA (*p* > 0.05). From the results shown in Table 3, it was found that the mean values of subjective well-being and its three dimensions in the medium and large exercise groups were significantly greater than those in the small exercise group (*p* < 0.01), and there was no significant difference in subjective well-being between large exercise volume group and medium exercise volume group (*p* > 0.05).

**Table 3:** The results of the variance analysis of the effect of physical exercise on the subjective well-being in college students.

Variable	SEV	MEV	LEV	*F*	*p*
GWB	44.06 ± 7.30	46.66 ± 6.88^**^	46.85 ± 7.48^###^	10.50	0.000
SIL	6.51 ± 1.86	7.02 ± 1.96^**^	7.24 ± 2.11^###^	8.24	0.000
MHM	17.71 ± 3.37	18.72 ± 2.85^**^	18.65 ± 3.05^##^	6.94	0.001
RT	19.84 ± 3.72	20.92 ± 3.27^**^	20.96 ± 3.61^##^	7.01	0.001

** SEV compared with MEV *p* < 0.01.

## SEV compared with LEV *p* < 0.01.

### SEV compared with LEV *p* < 0.001.

SEV, small exercise volume; MEV, medium exercise volume; LEV, large exercise volume; GWB, subjective well-being; SIL, satisfaction and interest in life; MHM, melancholy or happy mood; RT, relaxation and tension.

As shown in Table 4, first, with physical exercise as an independent variable, body image as a dependent variable, the regression coefficient of physical exercise was statistically significant (*β* = 0.22, *p* < 0.001). Second, with physical exercise and body image as an independent variable, self-esteem as a dependent variable, both the regression coefficient of physical exercise (*β* = 0.14, *p* < 0.001) and body image (*β* = 0.28, *p* < 0.001) were statistically significant. Third, with physical exercise, body image, and self-esteem as the independent variable, subjective well-being and its various dimensions as the dependent variables, the regression coefficient of self-esteem was statistically significant (*β* = 0.63, *p* < 0.001), both the regression coefficient of physical exercise and subjective well-being were not statistically significant (*p* > 0.05).

**Table 4:** Regression analysis of physical exercise, body image, self-esteem, and subjective well-being in college students (*N* = 671).

Outcome	Predictor(s)						*R*^2^	Physical exercise		Body image		Self-esteem	
	*β*	*T*	*β*	*T*	*β*	*T*	
Body image	0.22	5.75^***^					0.05
Self-esteem	0.14	3.73^***^	0.28	7.42^***^			0.11
GWB	0.02	0.51	0.05	1.58	0.63	19.90^***^	0.42
SIL	0.03	0.94	0.07	2.09^*^	0.48	13.61^***^	0.27
MHM	−0.01	−0.15	0.025	0.73	0.58	17.43^***^	0.35
RT	0.02	0.54	0.04	1.09	0.49	13.68^***^	0.25

GWB, subjective well-being; SIL, satisfaction and interest in life; MHM, melancholy or happy mood; RT, relaxation and tension.

^*^p < 0.05; ^***^p < 0.001.

As shown in Table 5, the PROCESS macro program was used for the mediation analysis, repeated sampling 5,000 times from the original data to calculate the 95% CI. If the 95% CI of the standardized path coefficient does not contain 0, it indicates that the mediating effect is significant. The direct effects of physical exercise on subjective well-being and its various dimensions all contain zero, indicating that physical exercise did not directly affect subjective well-being. From physical exercise through body image to subjective well-being, the mediating role of 95% CI was (−0.003, 0.028), the interval contains 0, which indicated that the mediating effect was not significant. From physical exercise through self-esteem to subjective well-being, the mediating role of 95% CI was (0.038, 0.141); and from physical exercise through body image, self-esteem to subjective well-being, the serial mediating role of 95% CI was (0.022, 0.058). The above two intervals did not contain 0, which indicated that the mediating effects were significant.

**Table 5:** The path and effect decomposition table of physical exercise on subjective well-being.

Effect	Path relationship	Effect size	Bootstrap *SE*	LLCI	ULCI
Total effect	PARS→GWB	0.151	0.038	0.076	0.226
	PARS→SIL	0.144	0.038	0.069	0.219
	PARS→ Energy	0.169	0.038	0.094	0.244
	PARS→MHM	0.116	0.038	0.041	0.192
	PARS→RT	0.124	0.038	0.049	0.199
Direct effect	PARS→GWB	0.016	0.031	−0.044	0.076
	PARS→SIL	0.032	0.034	−0.035	0.100
	PARS→ Energy	0.038	0.032	−0.025	0.101
	PARS→MHM	−0.005	0.032	−0.069	0.059
	PARS→RT	0.019	0.035	−0.049	0.087
Indirect effect	PARS→MBSRQ→GWB	0.011	0.008	−0.003	0.028
	PARS→SES→GWB	0.087	0.026	0.037	0.141
	PARS→MBSRQ→SES→GWB	0.038	0.010	0.021	0.058
Indirect effect	PARS→MBSRQ→SIL	0.016	0.011	−0.003	0.040
	PARS→SES→SIL	0.067	0.020	0.029	0.107
	PARS→MBSRQ→SES→SIL	0.029	0.007	0.016	0.044
Indirect effect	PARS→MBSRQ→MHM	0.005	0.009	−0.012	0.024
	PARS→SES→MHM	0.081	0.024	0.035	0.129
	PARS→MBSRQ→SES→MHM	0.035	0.009	0.019	0.054
Indirect effect	PARS→MBSRQ→RT	0.008	0.010	−0.009	0.029
	PARS→SES→RT	0.068	0.021	0.027	0.109
	PARS→MBSRQ→SES→RT	0.029	0.008	0.016	0.045

PARS, physical exercise; MBSRQ, body image; SES, self-esteem; GWB, subjective well-being; SIL, satisfaction and interest in life; MHM, melancholy or happy mood; RT, relaxation and tension; LLCI, lower confidence interval (95%); ULCI, upper confidence interval (95%).

## Discussion

### The Relationship Between Physical Exercise and Subjective Well-Being in College Students

This study assumes that physical exercise is positively correlated with the subjective well-being of college students. Data analysis also supports this hypothesis, which is consistent with Li and Liu (2014). Thus, hypothesis H1 is established. Besides, the study further concludes that physical exercise is positively correlated with all dimensions of the subjective well-being of college students. A previous study has shown that moderate and high-intensity aerobic exercise done three or more times a week contributes to reductions in negative emotions intervention (Liu, 2020). Xiao et al. (2017) showed that depression scores of students with small levels of exercise were significantly higher than those of middle and large levels of exercise, as indexed with the PARS-3. The research by Jiang and Zhu (1997) also found that medium to heavy exercise can help college students produce better health effects. These also suggest that only when the amount of activity reaches a certain level then it can affect subjective well-being. This study found that the scores of subjective well-being and its dimensions of college students in the medium and large exercise groups were higher than those in the small exercise group. The result further supports the view that “mid-to-high levels of exercise has a more positive effect on the subjective well-being of college students.”

### The Role of Body Image Between Physical Exercise and Subjective Well-Being in College Students

A positive body image is related to lower psychological problems, such as depression and social avoidance (Feng, 2005). Anxiety, depression, emotions, and interpersonal relationships are all important factors affecting subjective well-being (Chen et al., 2015). Therefore, researchers often use body image as the antecedent variable of negative emotions (Jing et al., 2016) and subjective well-being (Cash, 2004). As Borges et al. (2013) pointed out that the main predictors of happiness are appearance and physical satisfaction. Moreover, physical exercise can inhibit the formation of negative body image of college students. Furthermore, the Asci (2002) survey found that physical exercise and body image are significantly related, and people who take long-term physical exercise have better body image than those who do not exercise. Thus, this study deduces that the body image can play a mediating role between physical exercise and the subjective well-being of college students. However, the results showed that the mediating role of body image does not hold. Thus, hypothesis H2 is rejected.

### The Role of Self-Esteem Between Physical Exercise and Subjective Well-Being in College Students

Self-esteem is an important indicator of mental health. In many studies investigating the psychological benefits of exercise, self-esteem was used as an important indicator to measure the psychological benefits after exercise (Fang and Chen, 2016). Previous studies have shown that physical activity has many effects on the body and mind of an individual. Some related studies tend to believe that physical exercise has a certain positive effect on the self-esteem of people (Edward et al., 1997; Zhong et al., 2006; Qasim et al., 2014). Yu and Mao (2013) inferred that physical exercise might be an important factor affecting the development of self-esteem of students. A study (Fang, 2008) on 939 college students found that as long as they insist on exercising, no matter what type of exercise they do, they will improve their self-esteem. Furthermore, self-esteem is one of the most reliable predictors of subjective well-being (Diener, 1984; Diener and Diener, 1995). It is not difficult to conclude that self-esteem plays a “bridge” role between physical exercise and subjective well-being through previous research. The effect of self-esteem on physical exercise and subjective well-being also has been studied. Chen and Ji (2006) took high school students as the research object and found that physical exercise can not only directly affect the subjective well-being of high school students but also influence the subjective well-being of high school students through intermediary variables such as self-esteem, interpersonal relationship, and personality. This is consistent with the conclusion of this study. Hence, self-esteem plays an intermediary role between physical exercise in college students and subjective well-being. Thus, hypothesis H3 is established.

### The Role of Body Image and Self-Esteem Between Physical Exercise and Subjective Well-Being in College Students

Previous studies (Shin and Paik, 2003; Koronczai et al., 2013) affirmed the positive effect of body image on self-esteem, such as what Zeng and Huang (2001) pointed out in a review of body image: physical attributes of the human body like appearance, physical fitness, and health status. It is through the self-integration of the body into the overall self-concept. At the same time, this view is also evaluative. It will have a lasting impact on the self-esteem and self-confidence of people based on their social reference. This study further investigated the intermediary role of body image and self-esteem between physical exercise and the subjective well-being of college students. The results show that body image and self-esteem play a serial mediating role between physical exercise and the subjective well-being of college students. Thus, hypothesis H4 is established. And this result is understandable, people who take long-term physical exercise have better body image than those who do not exercise (Asci, 2002). It can be seen that physical exercise may contribute to the formation of positive body image of college students, and the positive cognition and evaluation of self-body may produce positive self-efficacy and high self-esteem (Guo et al., 2017), and then may affect their cognitive well-being and emotion such as satisfaction and interest in life, melancholy or happy mood, and relaxation and tension.

### Implications

COVID-19, as a public health event with strong infectivity and fast spread, has caused a certain psychological burden to the public (Huang and Zhao, 2020). As a special social group, college students have not yet fully matured physically and mentally and are in a period of the high incidence of psychological problems. In the major epidemic and other social life stress events, their mental health should be paid close attention to. This study clarified the relationship between physical exercise, body image, self-esteem, and subjective well-being in Chinese college students, providing ideas for the intervention of mental health of college students.

The determination of the relationship between physical exercise and subjective well-being suggests that we should cultivate long-term awareness of physical exercise in college students and encourage them to exercise at a medium to a high level every week.

Kou (2017) believed that perspective-taking has the function of reducing negative body image in adolescent individuals. And some researchers suggested that group counseling can effectively improve the self-esteem of individuals (Lin and Hong, 2020). The support for a serial mediation role of body image and self-esteem between physical exercise and subjective well-being provides new ideas for the intervention of mental health of college students. Teachers can try to improve the efficiency of the intervention of subjective well-being of college students through the cultivation of long-term physical exercise awareness, the ability of perspective-taking, and the comprehensive use of group counseling methods.

### Limitations and Future Research Directions

This study clarified the relationship between physical exercise, body image, self-esteem, and subjective well-being in Chinese college students, which had theoretical and practical implications. However, this study also has some limitations (1) owing to the use of cross-sectional studies, it was impossible to infer the causal relationship between variables. In the future, follow-up design and experimental studies can be used to test (2) Since the three universities selected in this study are all science and engineering universities, there are far more male college students than females in the sample obtained, so it failed to examine the influence of gender variables in the research variables. Future research should consider gender factors to make reasonable suggestions for college students of different genders.

## Conclusion

The present study is unique in examining the relationship between physical exercise, body image, self-esteem, and subjective well-being in a sample of Chinese college students. This study finds that students with the medium and high level of physical exercise have a higher score of subjective well-being than those with the low-level exercise. Self-esteem is established as mediators of physical exercise and subjective well-being, and the combination of body image and self-esteem is also established as a serial intermediary role between physical exercise and subjective well-being in college students. The confirmation of the path related to physical exercise and subjective well-being provides evidence for clarifying the relationship between physical exercise and subjective well-being. It provides a reference for the intervention of mental health of college students.

## Data Availability Statement

The original contributions presented in the study are included in the article/Supplementary Material, further inquiries can be directed to the corresponding author.

## Ethics Statement

The studies involving human participants were reviewed and approved by the Ethics Committee at the Chengdu Sports University. The patients/participants provided their written informed consent to participate in this study.

## Author Contributions

YS, S-YY, and H-DX: conceptualization, analysis, and writing—original draft preparation. YS and H-DX: methodology and software. YS and S-YY: resources and data curation. YS: writing—review and editing. All authors contributed to the article and approved the submitted version.

### Conflict of Interest

The authors declare that the research was conducted in the absence of any commercial or financial relationships that could be construed as a potential conflict of interest.
